# Neuronal Intranuclear Inclusion Disease: A Confirmed Case Report and Analysis of MRI Characteristics in Three Typical Cases

**DOI:** 10.2174/0115734056335449250407103447

**Published:** 2025-04-21

**Authors:** Jin Liu, Chuan Zhang, Jiwu Wang, Hanfeng Yang

**Affiliations:** 1 Department of Radiology, Jianyang Chinese Medicine Hospital, Chengdu, Sichuan 641400,China; 2 Department of Radiology, Affiliated Hospital of North Sichuan Medical College, Nanchong, Sichuan 637000, China; 3 Department of Radiology, Panzhihua Central Hospital, Panzhihua, Sichuan 617000, China

**Keywords:** Neuronal intranuclear inclusion disease, Magnetic resonance imaging features, Diffusion weighted imaging, Gene testing, Familial headache

## Abstract

**Objective:**

Neuronal Intranuclear Inclusion Disease (NIID) is a rare and clinically heterogeneous neurodegenerative disorder leading to diagnostic challenges. This study aims to investigate the clinical and characteristic radiological features of four adult female patients, offering insights into the clinical and radiological heterogeneity of NIID and its misdiagnosis potential.

**Case Representation:**

This case study presents a retrospective analysis of clinical data from four adult female patients, including one confirmed case and three with typical Magnetic Resonance Imaging (MRI) manifestations. The high signal intensity patterns on Diffusion-Weighted Imaging (DWI) and Fluid-Attenuated Inversion Recovery (FLAIR) sequences were reviewed in focus.

**Discussion:**

All four patients were adult females with common symptoms of NIID, such as recurrent headaches, cognitive decline, and autonomic dysfunction, accompanied by symptoms like vomiting, slowed responses, behavioral changes, and focal neurological symptoms. Genetic testing revealed a NOTCH2NLC gene mutation with GGC>113 repeats in one patient. Three patients from the same family presented with headaches, followed by vomiting and progressive unresponsiveness with two of them exhibiting abnormal behavior and one experiencing weakness and pain in the right limbs. Neurological assessments revealed peripheral neuropathy and intermittent confusion, among other manifestations. MRI features of all four patients were consistent with NIID, displaying high signals at the corticospinal junction on DWI and FLAIR sequences, with one case involving the vermis of the cerebellum.

**Conclusion:**

This case report enhances our understanding of NIID's diverse clinical phenotypes and the critical role of advanced MRI and genetic testing in its diagnosis. The core imaging feature of NIID is the high signal along the corticospinal junction on MRI, which, combined with NOTCH2NLC gene testing, can significantly enhance the early recognition and diagnosis of NIID. Therefore, this study deepens our understanding of the complex clinical phenotypes and imaging characteristics of NIID, providing crucial guidance for clinical practice.

## 1 INTRODUCTION

Neuronal Intranuclear Inclusion Disease (NIID) is a rare neurodegenerative disorder characterized by the presence of eosinophilic intranuclear inclusions in central, peripheral, and autonomic nervous system cells, as well as in visceral organs. The clinical spectrum of NIID is highly heterogeneous, encompassing cognitive impairment, Parkinsonian-like behaviors, peripheral neuropathy, cerebellar ataxia, tremor, gait instability, muscle rigidity, involuntary movements, muscle weakness, epilepsy, and headaches [1, 2]. Such a wide range of clinical phenotypes makes the diagnosis of NIID a formidable task. Historically, the definitive diagnosis of NIID depended largely on post-mortem brain pathology, which was time-consuming and limited in its applicability [3, 4]. Recent advances in diagnostic techniques, including skin biopsy and genetic testing for the NOTCH2NLC gene, have significantly transformed the diagnostic landscape for NIID [5]. In 2019, an expansion of the GGC repeat sequence in the 5' UTR of the NOTCH2NLC gene was associated with NIID, predominantly observed in individuals of Asian descent [5]. Despite these breakthroughs, the precise mechanisms connecting the GGC repeat expansion to the pathogenesis of NIID are not yet fully understood and warrant further research. The advent of advanced Magnetic Resonance Imaging (MRI) technology has unveiled characteristic imaging features of NIID, such as high signal intensity in bilateral cerebral white matter on T2, Fluid-Attenuated Inversion Recovery (FLAIR) sequences, and specific high-intensity signals at the corticomedullary junction on Diffusion-Weighted Imaging (DWI). However, these features may be absent in the muscle weakness and Parkinsonian syndrome subtypes of NIID, and DWI abnormalities may appear years after the onset of symptoms or even disappear as the disease progresses [6]. Therefore, the diagnosis and treatment of NIID require a comprehensive consideration of clinical manifestations, genetic testing, and advanced imaging techniques to improve diagnostic accuracy and timeliness. Thus, this case report presented a retrospective analysis of four adult patients, focusing on their clinical histories and MRI features. All four patients exhibited sporadic headaches, cognitive decline, and autonomic dysfunction. Upon genetic testing and imaging analysis, one was a confirmed NIID case, and three had typical NIID MRI manifestations. This case study will contribute to a better understanding of the clinical features of NIID and deepen our knowledge of this complex disease.

## 2 CASE ANALYSIS

We reviewed the medical records of four patients, including one confirmed case and three with typical MRI features. All MRI examinations were performed as routine clinical procedures using a 1.5 T scanner, with each patient receiving both DWI, T2, and FLAIR images. Two neuroradiologists independently analyzed the MRI data retrospectively, followed by a combined assessment.

### 2.1 Case 1

**Fig. (1):**
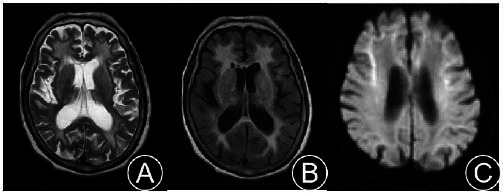
Cranial MRI: Symmetrical large patchy T2WI and FLAIR high signals in the corticomedullary junction, centrum semiovale, and corpus callosum of both cerebral hemispheres (**A**, **B**); DWI shows band-like high signals in the corticomedullary junction of both cerebral hemispheres, with high signals in the genu and splenium of the corpus callosum (**C**).

**Fig. (2):**
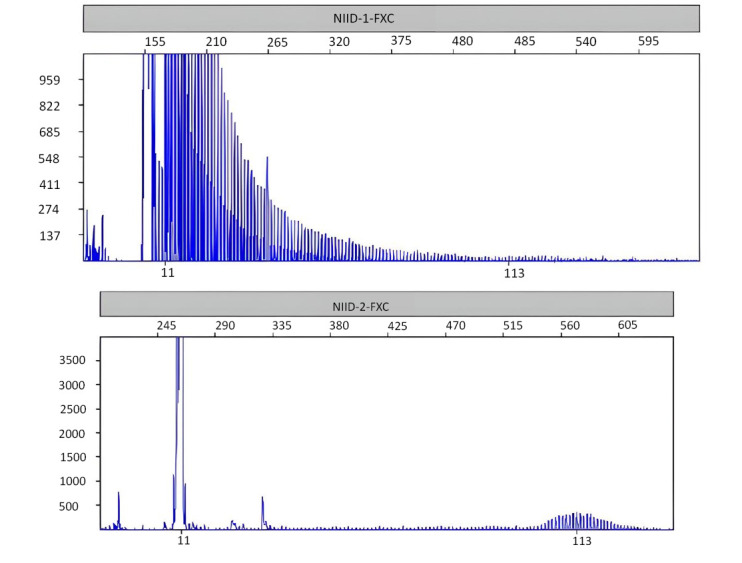
Gene Test: The NOTCH2NLC gene test showed GGC>113 repeats.

A 71-year-old Chinese female was admitted due to “poor appetite and vomiting for over 10 days, dizziness, fatigue, and unsteady walking for one week”. More than 10 days before admission, the patient presented a poor appetite and repeated vomiting after eating without any obvious cause. She exhibited dazed behavior, significantly reduced speech, lethargy, and poor sleep at night. One week before admission, she began experiencing dizziness, fatigue, and unsteadiness while standing and walking, with repeated falls during the course of the illness, which were not relieved by treatment. At the time of consultation, the patient's brain MRI symptoms were consistent with the imaging features of NIID (Fig. **1**), and NOTCH2NLC gene testing showed the presence of GGC>113 replicates (Fig. **2**).

### 2.2 Case 2 (Deceased)

**Fig. (3):**
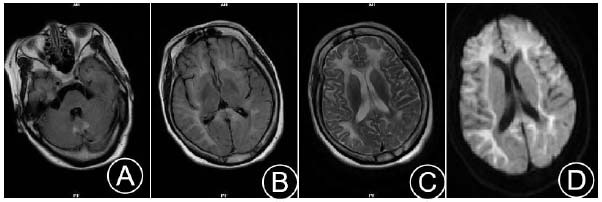
Cranial MRI: Symmetrical small patchy high signals in the cerebellar vermis on T2WI (**A**); symmetrical large patchy high signals in the corticomedullary junction, centrum semiovale, and the genu and splenium of the corpus callosum on FLAIR and T2WI (**B**, **C**); DWI shows band-like high signals in the corticomedullary junction of both cerebral hemispheres, with high signals in the genu and splenium of the corpus callosum (**D**).

A 48-year-old Chinese female was admitted due to “headache and vomiting for one day”. More than one day before admission, the patient experienced a sudden onset of head pressure pain without any obvious trigger, which intensified paroxysmally. During severe episodes, she suffered from nausea and non-projectile vomiting of stomach contents, accompanied by sweating and dizziness. Similar symptoms were repeated several times in the past without receiving treatment outside the hospital. At the time of consultation, the patient's imaging features were consistent with some of the typical MRI manifestations of NIID (Fig. **3**), such as symmetrical large patchy high signals at the cortical-medullary junction of the corpus callosum, the center of the semiovals, the knee, and the spleen, as well as banded high signals on DWI.

### 2.3 Case 3 (Daughter of Case 2)

**Fig. (4):**
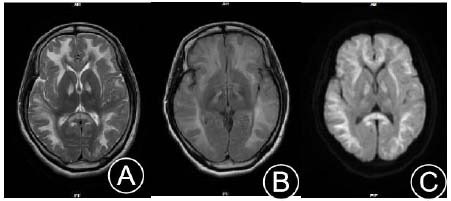
Cranial MRI: Symmetrical large patchy high signals in the basal ganglia, corticomedullary junction, centrum semiovale, and the genu and splenium of the corpus callosum on T2WI and FLAIR (**A**, **B**); DWI shows band-like high signals in the corticomedullary junction of both cerebral hemispheres (**C**).

A 27-year-old Chinese female was admitted due to a “headache accompanied by abnormal behavior for over 3 years, worsened with unresponsiveness for 9 hours”. According to the patient’s family, she began experiencing headaches more than 3 years ago after giving birth to her second child. The headaches were initially tolerable and affected the entire head but gradually became severe and unbearable, the exact nature of which was unknown. The headaches were followed by vomiting of stomach contents, general fatigue, and abnormal behavior, mainly characterized by intermittent incoherent speech and easy crying. The patient often self-medicated for pain relief, which provided temporary relief. The symptoms recurred and progressively worsened over time. More than 2 years ago, she sought medical attention for the pain and was diagnosed with “leukoencephalopathy” (Fig. **4**).

### 2.4 Case 4 (Niece of Case 2)

**Fig. (5):**
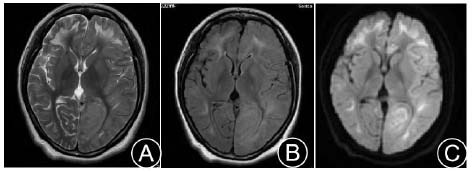
Cranial MRI: Symmetrical large patchy high signals in the corticomedullary junction and centrum semiovale of both cerebral hemispheres on T2WI and FLAIR (**A**, **B**); DWI shows band-like high signals in the corticomedullary junction of both cerebral hemispheres (**C**).

A 22-year-old Chinese female was admitted due to “episodic headache, right-sided limb weakness, and pain for over 4 years, with recurrence for 4 days”. Four years prior to admission, the patient received multiple medical consultations and was diagnosed with “mitochondrial encephalomyopathy” due to “headaches, weakness, pain in right-sided limbs,” and repeated episodes of the above symptoms (Fig. **5**).

## 3 DISCUSSION

Since the first confirmed case of NIID reported by Lindenberg *et al*. in 1968 [7], research on NIID has been ongoing. It was not until 2019, with the advent of NOTCH2NLC gene screening, that the causative factor was identified as the abnormal expansion of GGC repeat sequences in the 5’ UTR of the NOTCH2NLC gene [8], marking the beginning of the molecular diagnostic era for NIID. Current research indicates a correlation between the number of GGC repeats and the pathogenicity of NIID. A threshold of over 60 GGC repeats in the 5' UTR of the NOTCH2NLC gene is generally considered to be associated with the onset of the disease [9]. In this study, all four patients were adult females, and genetic testing of Case 1 revealed more than 113 GGC repeats. This high number of GGC repeats exceeded the threshold currently believed to be associated with disease onset, further supporting the correlation between GGC repeat sequences and the pathogenicity of NIID.

This provides evidence at the molecular level for the diagnosis of NIID and potential targets for the development of new therapeutic strategies. The study by Chen noted that GGC repeat expansions in the NOTCH2NLC gene were observed in NIID patients from some European and Far Eastern populations, suggesting that genetic background differences might lead to varying pathogenic mechanisms across regions [10]. This emphasizes the importance of considering the geographic and ethnic diversity in the genetic and clinical manifestations of NIID. Moreover, Chen *et al*. further suggested that the size of GGC repeat sequences is associated with a complex of disease phenotypes, which may range from asymptomatic to specific symptoms, such as autonomic dysfunction and visual abnormalities [9]. NIID, with its association with the NOTCH2NLC gene repeat amplification, calls for a deeper understanding of its genetic and clinical characteristics across different populations. Further research is necessary to elucidate the intricate relationship between genetic variations and the diverse clinical presentations of NIID, paving the way for more personalized and effective treatment approaches.

NIID exhibits a broad spectrum of clinical symptoms. Typically, NIID is classified into three clinical subgroups based on age of onset and disease progression: infants, adolescents, and adults [2]. Infantile-onset NIID is characterised by cerebellar manifestations of ataxia and dysarthria occurring before the age of 5 years; adolescent-onset NIID presents behavioral changes, followed by the development of pyramidal and cerebellar signs; and adult-onset cases are further differentiated into dementia-dominant and limb weakness-dominant phenotypes [5]. A significant research by Tai *et al*. on a Chinese cohort of 223 patients underscored the clinical diversity of NIID and endeavored to formulate a classification system [4]. The study indicated that about 65% of the patients were female, with an average onset age of approximately 57 years. A recent comparative study found significant differences between Japanese and non-Japanese (*e.g*., European) cases in terms of age of onset, clinical presentation, and diagnostic approach. Compared to non-Japanese cases, Japanese cases often presented with cognitive impairment as the primary symptom, had an older age of onset, and exhibited a higher rate of diagnosis through skin biopsy [11]. Despite the scarcity of literature on NIID in infants and adolescents, existing findings suggest that the continuous development of the infant nervous system in these age groups may lead to unique clinical symptoms, making diagnosis and research challenging [12, 13]. Collectively, these studies underscore the clinical and genetic heterogeneity of NIID, particularly across different age groups and geographic populations.

Our study presents a detailed analysis of four adult female patients diagnosed with NIID, each exhibiting a range of clinical symptoms that corroborate the diverse nature of the disease [14, 15]. It has been reported that many NIID patients, especially those with increasing age, typically exhibit autonomic dysfunction, such as vomiting, gastrointestinal dysfunction, arrhythmia, *etc* [2, 16]. It was consistent with the symptoms of the four patients. Additionally, two patients experienced vomiting; one exhibited mental abnormalities, and another presented with muscle weakness. NIID has been recognized as the second most prevalent adult-onset hereditary white matter disease [17], a fact that was evident in case 3. Furthermore, the association of NIID with mitochondrial encephalomyopathy was mirrored in the clinical presentation of case 4. This observation underscores the complexity of NIID and its overlap with other neurodegenerative conditions [18]. This observation underscores the complexity of NIID and its overlap with other neurodegenerative conditions. The presence of these symptoms and the overlap with mitochondrial encephalomyopathy in our patients emphasize the need for a comprehensive clinical approach and the importance of considering NIID in the broader context of neurodegenerative diseases. Furthermore, Feng *et al*. expanded the understanding of NIID impacts by identifying retinal structural and functional abnormalities as part of the disease's clinical spectrum, which are distinct from those observed in traditional retinal pathologies [19, 20]. This finding is particularly significant as it highlights the potential risk of glaucoma in NIID patients with pupil constriction, adding a new dimension to the disease's ocular implications. Further exploration of the genetic basis, pathophysiological mechanisms, and intersections with other neurological diseases in NIID is needed for future research.

The first recorded familial NIID case in Korea suggested the prevalence of both familial and sporadic NIID cases [20]. Cases 2-4 in our study belong to the same family and initially presented with headache symptoms. Headache, as a new early phenotype of NIID, is particularly important in familial cases. This feature has been rarely mentioned in the previous literature, so this finding fills the gap in early diagnostic markers of NIID. At the same time, the unique clinical manifestations of familial NIID patients suggest that attention should be paid to the genetic background of such patients in order to identify the potential risk earlier. This observation is significant as Peng *et al*. found that unexplained headache is a new early phenotype of NIID in sporadic and familial NIID [5]. In familial NIID cases, headache is not only the common early symptom, but also accompanied by other specific signs, such as muscle weakness, sensory disturbances, and juvenile onset. These phenotypic features provide new insights into the inheritance pattern of NIID. This may lead to a more complex clinical presentation. Therefore, in patients with a family history, comprehensive genetic testing and imaging evaluation should be performed in order to identify the disease earlier and intervene. It has also been reported that familial NIID cases are more likely to be associated with muscle weakness, sensory disorders, and adolescent onset [2]; this was particularly evident in cases 3 and 4 of our study. Fujita *et al*. also emphasized the phenotypic diversity of familial NIID, including muscle weakness, dementia, and Parkinson's syndrome [21]. Wu *et al*. confirmed the NIID diagnosis in two brothers through a skin biopsy, which revealed characteristic intranuclear inclusions with strong positivity for P62 and ubiquitin antibodies [17]. Fan *et al*. suggested in clinical practice that when NIID is diagnosed in a family member, MRI scans and genetic testing should be considered for other symptomatic family members, with vigilance for the possibility of NIID [22]. While our case provides preliminary evidence that suggests certain typical presentations in familial NIID, it is clear that more data are warranted to further investigate the association between headache or other clinical symptoms and familial NIID and the full range of NIID presentations. By expanding our knowledge of the presentations of the disease, we can refine diagnostic criteria, optimize clinical management, and ultimately improve the quality of care for patients affected by NIID.

MRI of the brain in adult-onset NIID patients shows characteristic features, such as high-intensity signals at the corticomedullary junction on DWI, known as the “band sign” [23, 24]. This imaging signature is a critical diagnostic marker for NIID. This study confirmed the high signal at the cortico-medullary junction as the hallmark imaging feature of NIID and, combined with the NOTCH2NLC gene detection results, provided clinicians with an efficient method for early diagnosis. This method not only improves the accuracy of diagnosis but also shortens the time to diagnosis, which helps to take timely interventions and improve the prognosis of patients.

Clinical manifestations of NIID are diverse and can result in misdiagnosis, especially in cases of acute and chronic seizures, which may be erroneously classified as encephalitis, epilepsy, vascular encephalopathy, or Parkinson's disease [25]. To address this challenge, the role of MRI in NIID diagnosis has been emphasized, with Sugiyama *et al*. highlighting characteristic MRI features, such as FLAIR high signals in the cerebellar folia and middle cerebellar peduncles and the high signals at the corticomedullary junction, which are critical diagnostic indicators on Diffusion-Weighted Imaging (DWI) and closely related to pathological changes in the skin and brain tissue [26]. Zhang *et al*. provided further refinement of the MRI characteristics of NIID through quantitative analysis, and proposed the “crested cockscomb sign” to describe the DWI high signals at the corticomedullary junction, noting the “T2WI-DWI spatial distribution mismatch”, where the lesion distribution is more extensive on T2WI [27]. These features, accompanied by reduced gray and white matter volumes and increased ADC values, especially in the frontal and parietal lobes and internal capsule regions where damage correlates with the course of the disease, offer unique imaging markers for NIID. The spatial distribution differences of T2WI-DWI provide a unique perspective to study the disease process. Through quantitative analysis of these imaging features, the severity and development trend of the disease can be more accurately evaluated so as to guide the selection of personalized treatment plans. Tokumaru *et* *al*. confirmed NIID cases in a Singaporean cohort, identifying DWI abnormalities in the subcortical U-fiber region as key for disease recognition [23]. By screening patients with characteristic MRI features and confirming the diagnosis through skin biopsy, they demonstrated that combining MRI imaging with skin biopsy is crucial for identifying NIID, especially in dementia patients. These studies underscore the core value of MRI imaging, particularly DWI, in NIID diagnosis. In our study, symmetrical T2WI and FLAIR hyperintensities were observed in key areas, such as the corticomedullary junction, semiovale center, and corpus callosum, in all four patients. DWI also showed banding hyperintensities at the corticomedullary junction, providing favorable evidence for the imaging diagnosis of NIID. The specific imaging manifestations of NIID on MRI, particularly on DWI, can effectively aid disease recognition and reduce misdiagnosis rates, complemented by skin biopsy and genetic testing for further confirmation, enhancing early and accurate diagnosis awareness among clinicians.

Limitations of this study include the lack of skin biopsy for Case 1 and diagnosis of Cases 2-4 based on typical MRI imaging features and familial context. Additionally, the sample size was small.

The clinical manifestations of NIID are highly diverse and similar to various diseases, complicating its diagnosis. Our case report, detailing four adult female patients, underscores the pivotal role of advanced MRI in identifying NIID. The sensitivity of MRI in revealing characteristic high signals at the corticomedullary junction on DWI, such as the 'subcortical ribbon sign,' 'subcortical flame sign,' 'saw-tooth sign,' or 'crested cockscomb sign', provides critical diagnostic insights. These imaging biomarkers, along with genetic testing results showing more than 113 GGC repeats in case 1, were instrumental in confirming the diagnosis. However, the report also notes that early-stage patients might not exhibit these typical imaging features, underscoring the necessity for a comprehensive diagnostic approach that integrates clinical symptoms and FLAIR imaging signals from regions outside the frontal lobe, such as the cerebellar vermis, middle cerebellar peduncles, and corpus callosum. As a new imaging marker, the hyperintensity of the cerebellar vermis suggests that it may be related to specific neuropathological mechanisms. An in-depth study of this feature will help to reveal the specific mechanism of cerebellar dysfunction in NIID and provide theoretical support for the development of targeted treatment. The case report advocates for an integrative diagnostic strategy that combines MRI imaging with genetic testing, especially focusing on the GGC repeat expansion analysis within the NOTCH2NLC gene. The genetic variants and clinical phenotypes of patients with familial NIID can be more comprehensively evaluated. For example, biallelic GGC repeat expansions may lead to more complex clinical manifestations. At the same time, the combination of imaging and genetic testing can better reveal the impact of these variants and provide a basis for personalized treatment. It also calls for increased sample sizes in future research to enhance the understanding of the clinical, imaging, and genetic heterogeneity of NIID. Utilizing a multifaceted approach, including skin biopsy and multiparametric MRI techniques, will be essential for improving diagnostic rates and exploring the full spectrum of the manifestations of NIID.

## CONCLUSION

In conclusion, our case report contributes to the growing body of evidence that supports the use of advanced diagnostic techniques in NIID, facilitating more accurate and timely patient management. The findings also highlight the necessity for continued research to explore the full spectrum of the clinical presentations of NIID and to develop personalized treatment strategies for this complex disease.

## Data Availability

All image data utilized have been verified to ensure authenticity and reliability.

## LIST OF ABBREVIATIONS

NIID: Neuronal Intranuclear Inclusion Disease
FLAIR: Fluid-Attenuated Inversion Recovery
DWI: Diffusion-Weighted Imaging

## STANDARDS OF REPORTING

CARE guidelines were followed.
